# SCOPE: A Spatial Co-registration and Omics Pipeline for Comparative Mass Spectrometry Imaging Data Analysis

**DOI:** 10.34133/csbj.0237

**Published:** 2026-10-26

**Authors:** Dingchang Shi, Emily MacFarlane, Berin A. Boughton, Paul J. Donaldson, George Guo, Angus C. Grey

**Affiliations:** ^1^ Department of Physiology, School of Medical Sciences, Faculty of Medical and Health Sciences, University of Auckland, Auckland, New Zealand.; ^2^ La Trobe Institute for Sustainable Agriculture and Food, La Trobe University, Melbourne, Victoria, Australia.

## Abstract

Spatially resolved omics technologies have transformed our ability to map molecular organization within tissues. However, the absence of anatomically consistent spatial units across biological replicates fundamentally limits cross-sample comparison and rigorous population-level inference at high spatial fidelity. Current approaches rely either on segmentation-dependent region definitions, which risk obscuring fine-scale molecular heterogeneity, or on analyses confined to individual specimens without cross-replicate spatial correspondence. Here, we introduce SCOPE, a spatial co-registration and omics pipeline that integrates deformable image co-registration with a shared spatial reference, HexBin, to establish anatomically homologous spatial units across samples. By enabling segmentation-free comparison while preserving high spatial fidelity, SCOPE maintains intrinsic molecular heterogeneity and supports statistically robust cross-replicate comparison. Application of SCOPE to an *ex vivo* bovine lens model of oxidative stress reveals region- and microdomain-specific remodeling of glutathione metabolism that is obscured by whole-tissue averaging or coarse segmentation. High-spatial-fidelity mapping identifies anterior–posterior asymmetries and localized vulnerabilities within lens fiber cell populations and enables relative quantitative evaluation of the thiol-based antioxidant therapeutic *N*-acetylcysteine amide. SCOPE advances spatial omics beyond segmentation-dependent and single-section descriptive analyses toward reproducible cross-sample quantitative inference, providing a scalable foundation for high-spatial-fidelity analysis across biological tissues.

## Highlights

• SCOPE is a workflow for anatomically aligned, cross-sample matrix-assisted laser desorption/ionization mass spectrometry imaging comparison.
• The “HexBin” framework preserves tissue microdomain heterogeneity across biological replicates.
• Region-specific lens redox responses to oxidative stress and therapy are spatially mapped.

## Graphical Abstract

**Figure:**
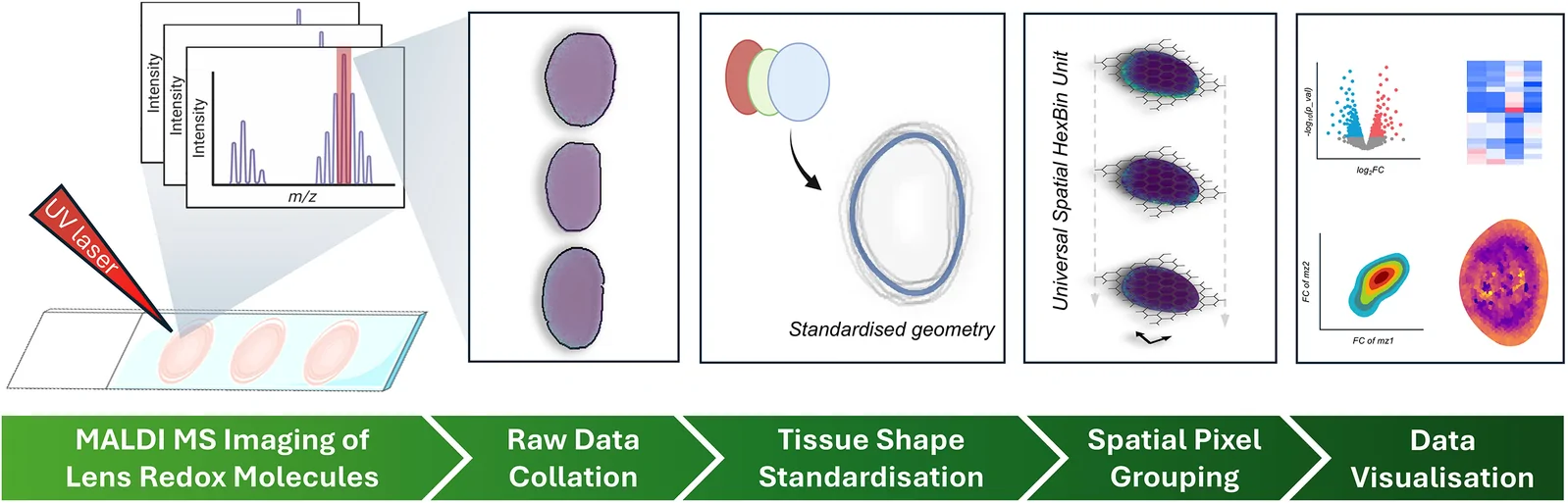

## Introduction

Matrix-assisted laser desorption/ionization mass spectrometry imaging (MALDI-MSI) has emerged as a powerful platform for spatially resolved molecular analyses, enabling simultaneous visualization and relative quantification of metabolites, lipids, proteins, peptides, pharmaceuticals, and other biomolecules directly from tissue sections at near-cellular resolution [1–7]. For example, MALDI-MSI has been used to visualize intratumor metabolic heterogeneity in the gastric cancer microenvironment [8], track lipid alterations in neurodegeneration [9,10], and understand glucose uptake and metabolism in the ocular lens [11]. This capability allows for the interrogation of tissue biochemistry *in situ*, capturing subtle spatially localized distributions associated with physiological and pathological processes. As instrument performance and data reproducibility have improved, MALDI-MSI has become increasingly high throughput, enabling the routine acquisition of large numbers of biological replicates. In parallel, recent advances in computational spatial omics have improved automated medical image preprocessing, multimodal data integration, and reproducible analysis workflows, facilitating increasingly sophisticated interrogation of complex spatial molecular datasets [12–14]. Despite greater sample throughput, robust spatial comparison across replicates remains challenging. Inherent differences in tissue section dimensions, orientation, and morphology disrupt pixel-to-anatomy correspondence across samples, compromising the reliability of high-spatial-fidelity comparisons without appropriate preprocessing.

Current approaches to spatial analysis of MALDI-MSI data often rely on either arbitrary segmentation or unsupervised clustering of pixels based on spectral similarity to delineate regions of interest (ROIs) that correspond to tissue areas with different anatomical or pathological labels [4–6,15–23]. Both strategies sacrifice much of the intrinsic spatial resolution of MALDI-MSI by grouping neighboring pixels into larger regions, risking the effective smoothing of local biochemical heterogeneity. This averaging process can obscure fine-scale molecular features and mask biologically relevant spatial differences. An alternative computational strategy is direct sampling from each tissue section region, which treats each pixel as an independent observation, using the total pixel count as the statistical sample size for group comparisons [9,24–26]. Although this approach preserves spatial detail, it ignores biological replicates and risks overestimating significance by inflating statistical power. As a result, it may bias findings toward individual outlier sections rather than population-level effects. To fully exploit the relatively high spatial resolution of MALDI-MSI while preserving the ability to incorporate multiple biological replicates into subsequent statistical analyses, spatial co-registration of tissue sections is essential. Existing methods align MSI data either to other imaging modalities obtained from the same section [27–29] or to adjacent sections from a single specimen [18,30,31]. Such intrasample registration approaches support high-fidelity visualization of molecular organization within individual specimens but lack a shared anatomical coordinate framework for cross-replicate comparison. Consequently, high-spatial-fidelity analyses are largely confined to single samples, limiting reproducibility and precluding rigorous population-level inference. Addressing this challenge requires establishing anatomically homologous spatial units across biological replicates. Furthermore, preserving fine-scale molecular heterogeneity without reliance on segmentation is essential to minimize loss of spatial information. In this context, we use the term “spatial fidelity” to refer to the preservation of anatomically corresponding spatial relationships across biological replicates following computational co-registration, rather than the intrinsic spatial resolution of the imaging system [32]. While spatial resolution determines the smallest feature that can be resolved during image acquisition, high spatial resolution alone does not ensure reliable anatomical correspondence across repeated measurement of independent biological samples, which is essential for comparative spatial analysis. Together, these considerations underscore the need for a computational framework that integrates image co-registration with a common spatial reference system, enabling anatomically consistent, high-spatial-fidelity comparisons across multiple samples.

In this study, we present SCOPE, an integrated spatial co-registration and omics pipeline for MALDI-MSI, providing high-fidelity, anatomically consistent comparisons of molecular distributions across biological replicates. By combining robust spatial co-registration with the construction of a common spatial reference, this workflow preserves fine-scale molecular heterogeneity while enabling population-level statistical analysis. We apply this framework to the ocular lens (Fig. S1), utilizing samples exposed to oxidative stress through hyperbaric oxygen (HBO) treatment, a well-established experimental model of lens aging [33,34]. This approach assesses the oxidative-stress-induced spatial alterations in glutathione (GSH) metabolism, the primary antioxidant system in the lens, relative to control tissue. This biological model is particularly valuable for investigating molecular changes that precede age-related nuclear (ARN) cataract formation [35,36], which is the most common form of cataract and is characterized by opacification of lens tissue in the central lens region. The MALDI matrix *N*-(1-naphthyl)ethylenediamine dihydrochloride (NEDC) was utilized to allow the detection of multiple antioxidant-related metabolites [37] that are known to change in the lens under oxidative stress conditions such as HBO treatment [33]. Furthermore, using *N*-acetylcysteine amide (NACA) as a pharmacological intervention against oxidative stress, we demonstrate region-specific evaluation of therapeutic modulation with our computational framework. Together, this strategy distinguishes localized metabolic alterations from tissue-wide effects and establishes a generalizable framework for anatomically consistent, high-spatial-fidelity comparison of metabolic phenotypes across biological cohorts.

## Materials and Methods

### Chemicals, reagents, and tissues

All chemicals and reagents were purchased from Sigma-Aldrich (MO, USA). NACA was obtained from Nacuity Pharmaceuticals Inc. (TX, USA). All solvents used were of high-performance liquid chromatography (HPLC) grade. Bovine eyes were obtained from the Auckland Meat Processing Plant (Auckland, New Zealand) approximately 4 h post-mortem and transported to the University of Auckland at room temperature.

### Lens incubation

Bovine lens dissections were performed under aseptic conditions in a sterile fume hood, with 70% ethanol used to minimize bacterial contamination. Lenses were carefully inspected for opacities, blemishes, cataracts, or yellowing, and any compromised lenses were excluded. Whole lenses were incubated in Gibco Medium 199 (M199, Thermo Fisher Scientific, MA, USA) in either the absence or presence of 10 mM NACA for 24 h prior to hyperbaric exposure. Oxidative stress was induced by exposing lenses to HBO (100 bar, 37 °C) for 15 h, either without NACA (HBOc) or with NACA supplementation (HBOn), while hyperbaric nitrogen (HBN; 100 bar, 37 °C) served as pressure control under the same conditions, without (HBNc) or with NACA (HBNn). Incubation media were refreshed immediately before hyperbaric exposure, allowing direct comparison of oxidative stress and hyperbaric pressure effects, with and without drug intervention.

### MALDI-MSI tissue preparation

Frozen bovine lenses were mounted onto pre-chilled chucks with Cryomatrix compound (Thermo Fisher Scientific, MA, USA). Axial cryosections (20 μm) were prepared using a cryostat (Leica 3050S, Leica Biosystems, Nussloch GmbH, Germany) and immediately transferred to pre-chilled, carbon-tape-coated glass slides (ProSciTech, Kirwan, Australia). Once all sections were mounted, slides were briefly warmed in the hand to bring them to room temperature and stored in a vacuum desiccator until use.

### Matrix application and MALDI-MSI

Matrix and internal standard solutions were applied with TM-Sprayer (HTX Technologies, NC, USA) coupled to an HPLC pump (LC-20AD, Shimadzu, Japan). NEDC (7 mg/ml in 90% MeOH) and 3-*O*-methyl-D-glucose (500 μM) were deposited under optimized conditions: solvent flow rate 0.15 ml/min, nozzle speed 700 mm/min, nozzle temperature 70 °C, track spacing 2 mm, drying time 0.5 min, and 4 passes [38]. After deposition, slides were stored in a vacuum desiccator at room temperature until analysis.

All MALDI-MSI experiments were performed on a Bruker timsTOF fleX MALDI-2 instrument equipped with a microGRID (Bruker Scientific Instruments, MA, USA). Data were acquired in negative ion mode over an *m*/*z* range of 50 to 1,000 using a laser power of 80% with 500 laser shots per pixel at a frequency of 10 kHz. A custom single laser spot was used with BeamScan enabled across a raster width of 71 × 71 μm, resulting in a final pixel size of 75 × 75 μm. These acquisition parameters ensured consistent ionization and spatial resolution across all samples.

### Data preprocessing and metabolite annotation

Untargeted spatial metabolomics datasets derived from MALDI-MSI were first loaded into SCiLS Lab software (Version 2025b Core, SCiLS GmbH, Germany) to check image quality and assess overall data integrity and then converted to .imzML format, normalized by the total ion count approach. The .imzML files were imported into R scripts using the Cardinal package [39,40]. Data preprocessing was adapted from the HiT-MAP package [6], with peak alignment at 15 ppm and exclusion of peaks with absolute intensity less than 5. Sections were reorientated where necessary to ensure consistent orientation, with the lens anterior surface oriented to the right. Using the “pixel_convolution()” function, the spatial resolution was down-sampled to 150 μm by averaging molecular intensities from 4 adjacent pixels acquired at a 75-μm resolution, thereby improving computational efficiency while retaining spatial detail. Off-tissue pixels were identified using a uniform manifold approximation and projection (UMAP)-based clustering approach, which distinguished off-tissue regions, primarily corresponding to areas of cracked tissue that were mainly covered by matrix only in the tissue sections (n_neighbors = 100 and min_dist = 0.1) [20]. These pixels were retained during the spatial co-registration process but were excluded from downstream HexBin-based quantitative and statistical analyses, such that only on-tissue pixels contributed to the final comparisons. Annotations of *m*/*z* features were made using a combination of accurate mass (15-ppm window), reference to previous on-tissue tandem mass spectrometry (MS/MS) [41], orthogonal liquid chromatography–MS/MS [42], and isotopic peak scoring [6], resulting in both high-confidence annotations and others with relatively lower confidence.

### Whole-tissue and segmentation-based low-spatial-fidelity analysis via SCOPE

To capture both global and spatially localized molecular changes, SCOPE analysis was conducted using whole-tissue averaging and distance-based segmentation approaches. Whole-tissue molecular intensities were averaged across the entire section, providing a global comparison between exposure groups, which was assessed using a *t* test. To resolve spatial heterogeneity within the tissue, arbitrary segmentation was applied based on the distance of each pixel from the lens center, as described by Shi *et al.* [15]. Segmentation-based comparisons were visualized using volcano plots to highlight significant molecular changes, heatmaps to assess intragroup variability, and radar plots to summarize mean intensities across exposure groups.

### High-spatial-fidelity analysis at the HexBin level via SCOPE

SCOPE performs high-spatial-fidelity analysis across multiple biological samples through tandem co-registration and HexBin discretization. Lens sections lack clearly identifiable anatomical landmarks; therefore, co-registration was based on their outer boundaries (geometries). All samples were aligned to a standardized lens geometry derived from high-resolution sagittal images, with anterior and posterior surfaces fitted to aspheric equations and the equatorial contour smoothed to ensure continuous curvature (“Model_lens.csv” in GitHub) [43]. Experimental section boundaries were extracted using the “find_edge_m()” function.

Co-registration began with a rescaling step, in which the geometries of the experimental sections and the template were centered at the origin (0, 0) and normalized to a Frobenius norm of 1 [44]. Tilt correction was then performed to align points from the experimental sections to the template geometry using the iterative closest point (ICP) algorithm, followed by pixel relocation via the barycentric deformation model implemented in the “align_to_model()” function. Here, the barycentric formulation is used as a geometric interpolation framework for deformable image co-registration. These procedures enabled consistent spatial alignment across samples.

Specifically, for each sample, the outer boundary was represented by a set of experimental edge points.

$$E_{i}=\left(x_{E_{i}},y_{E_{i}}\right)$$ (1)

The corresponding boundary points from the model geometry were matched using the ICP algorithm.

$$M_{i}=\left(x_{M_{i}},y_{M_{i}}\right)$$ (2)

This matching established a transformation that maps each experimental boundary point onto the model boundary (Fig. S4a (iii)).

Each interior pixel was denoted

$$p=\left(x_{p},y_{p}\right)$$ (3)

and, after boundary alignment, was assigned to a triangular domain defined by 2 adjacent experimental edge points $E_{1}$, $E_{2}$, and the lens center at the origin (0, 0). Within the triangle, the position of the pixel can be expressed in barycentric coordinates.

$$\lambda =\left(\lambda_{1},\lambda_{2},\lambda_{3}\right)$$ (4)

These coefficients are obtained by solving

$$\left[\begin{array}{ccc} x_{E_{1}}\; & x_{E_{2}} & 0\\ y_{E_{1}} & y_{E_{2}} & 0\\ 1 & 1 & 1 \end{array}\right]\left[\begin{array}{c} \lambda_{1}\\ \lambda_{2}\\ \lambda_{3} \end{array}\right]=\left[\begin{array}{c} x_{p}\\ y_{p}\\ 1 \end{array}\right]\;$$ (5)

These barycentric weights were then applied to the corresponding model triangle defined by the matched model edge points $M_{1}$, $M_{2}$ and the origin, computing the standardized reference-space location.

$$P=\lambda_{1}M_{1}+\lambda_{2}M_{2}+\lambda_{3}0$$ (6)

This procedure ensures that every interior pixel in the experimental image is smoothly and consistently projected onto the standardized model lens geometry based solely on the deformation guided by the matched edge boundaries.

After pixel relocation, HexBin indices were generated using the “hexbin()” function with the argument “xbins = 32”. The HexBin size is a user-defined parameter and can be adjusted through the “xbins” argument according to the spatial resolution and characteristics of the dataset. In the present study, xbins = 32 was selected based on our previous work with bovine lens MALDI-MSI datasets acquired at a 150-μm spatial resolution, providing a level of spatial averaging comparable to the weak denoising function implemented in SCiLS Lab while reducing pixel-level noise and preserving the anatomical spatial features of the lens [15]. The mean spectrum for each HexBin in individual samples was computed using the “HexBin_Signal()” function, and the average spectrum (average HexBin dataset) across 3 biological replicates within each exposure group was obtained using the “Average_HexBin_intensity()” function.

### High dimensionality reduction, clustering, and spatial mapping

For the initial comparison between HBNc and HBOc exposure groups, spatial clustering was performed in 2 steps on the average HexBin dataset, namely, dimension reduction and clustering based on the reduced dimensions. To reduce the dimensionality of the feature space while preserving critical information, “umap()” in the uwot package was employed with the arguments n_neighbors = 100 and min_dist = 0.5 [45]. Subsequently, *k*-means clustering was applied to the UMAP embedding using the “kmeans()” function in R to generate 6 clusters of HexBins. These clusters were projected to the aligned lens through HexBin labels for region annotation. UMAP_2 coordinates and spatial clusters of the HBNc group were used as the spatial reference for the spatial analysis at the HexBin level and mapping the spatial orientation of the HexBins in the dimensionality reduction of all exposure groups for the later NACA treatment study.

### Spatial analysis at the HexBin level

The HexBin-based statistical framework was applied to quantify and visualize spatial molecular alterations across samples. Specifically, the intensity values from each HexBin of individual samples were compared between experimental groups using 2-sample *t* tests. To account for multiple comparisons, *P* values were adjusted using the Benjamini–Hochberg false discovery rate (FDR) procedure across all tested *m*/*z* features and HexBins. Statistical significance was defined as an FDR-adjusted *P* value <0.1. The resulting fold-change and significance maps were then projected onto the standardized lens geometry to visualize the spatial distribution of molecular alterations. The spatial fold change for each *m*/*z* feature was computed using the “HexBin_FC_calculation()” function, while the spatial absolute intensity difference was derived using “HexBin_diff_calculation()”. These computed metrics were subsequently projected onto the aligned lens architecture via “Project_change_to_tissue()”, enabling the visualization of molecular changes in their anatomical context.

To explore spatial molecular relationships, spatial HexBin-level fold-change magnitude correlation analysis was performed using “Correlation_plot()”, which depicts the density distribution of fold changes across HexBins between any 2 *m*/*z* highlighting the covariation across HexBin. Furthermore, the fold-change heatmap, generated using “Plot_FC_HeatMap()”, provided a comprehensive spatial overview of molecular alterations between any 2 exposure groups, facilitating the identification of localized metabolic or compositional shifts within the lens tissue.

## Results

### A computational workflow for spatial alignment and validation of MALDI-MSI lens metabolomics

To address the potential loss of spatial resolution in standard MALDI-MSI data analysis workflows introduced by regional segmentation, we developed a new workflow that preserves fine spatial fidelity to accurately detect local metabolic alterations across tissue replicates. The workflow integrates all steps, ranging from the import and preprocessing of raw .imzML data from biological replicates to spatial registration, quantitative analysis, and visualization within this automated pipeline (Fig. 1). SCOPE utilizes the R package HiT-MAP for the preprocessing of .imzML data (peak alignment, peak filtering, normalization, image rotation, etc.) [6]. To further improve data quality, the framework also incorporates a systematic strategy for managing off-tissue regions, a critical step in MSI data interpretation (Fig. S2a to d) [21]. Specifically, a UMAP-based approach to determine the off-tissue regions is incorporated within the pipeline to ensure that only biologically relevant pixels are retained (Figs. S2b and c and S3a). This allows subsequent analyses to focus exclusively on genuine tissue-derived metabolic signals (Fig. S3b).

**Fig. 1.**
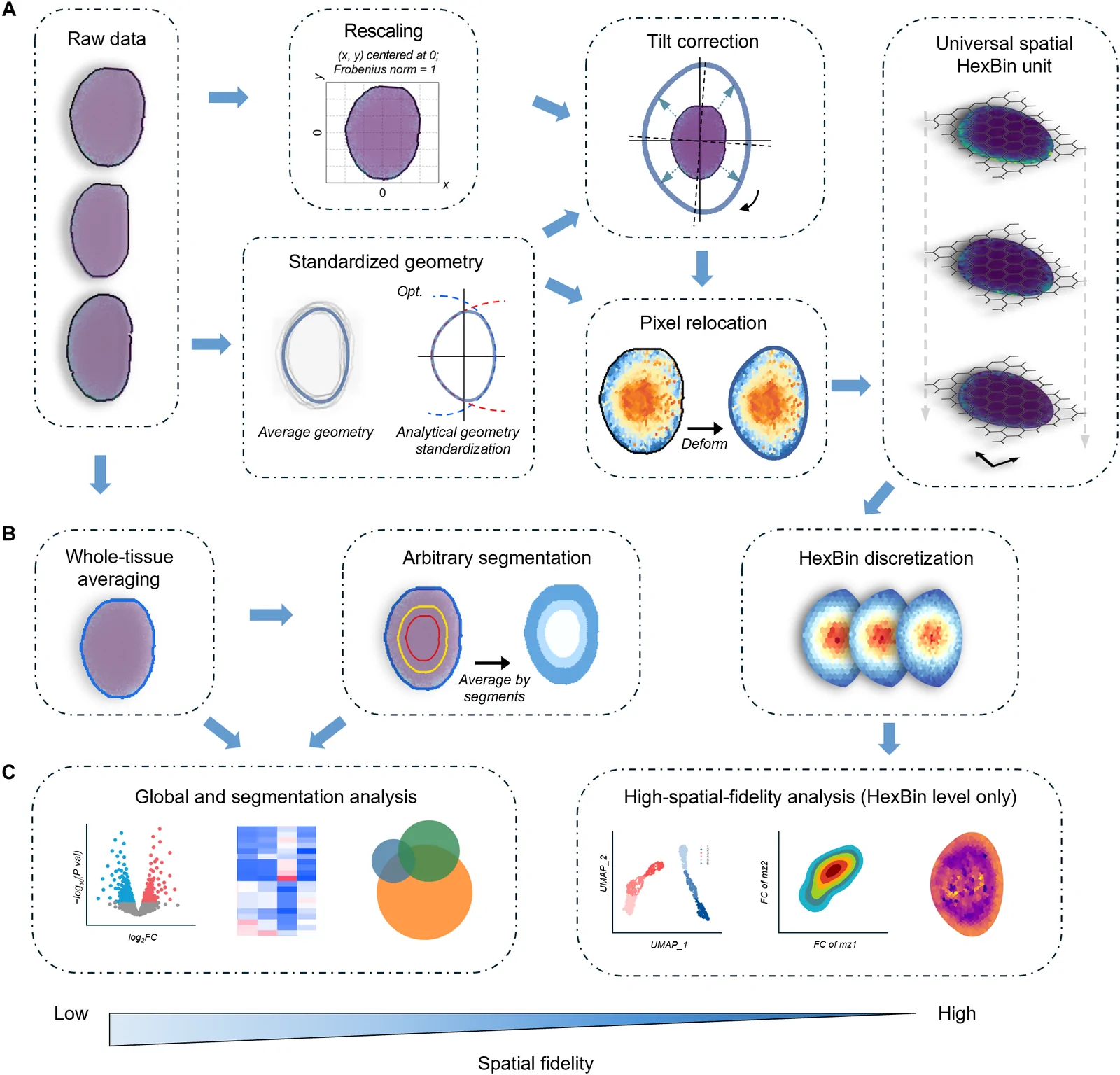
A schematic of the experimental workflow and pipeline of spatial metabolomic analysis of bovine lenses using matrix-assisted laser desorption/ionization mass spectrometry imaging (MALDI-MSI). (A) Overview of the SCOPE workflow for high-spatial-fidelity cross-sample analysis. Raw tissue sections with variable shapes and sizes are rescaled (centered at mean = 0 and normalized to Frobenius norm = 1) and aligned to a standardized tissue template. The template is derived either from the average geometry of all samples or from analytical geometry standardization. Rigid tilt correction is performed by rotating experimental geometries and establishing point correspondences to the template using the iterative closest point (ICP) algorithm. These correspondences are used to perform barycentric deformation, relocating pixels from raw coordinates into a common standardized coordinate space. A universal spatial HexBin discretization is then applied, enabling spatially matched comparisons across multiple biological samples. (B) SCOPE enables multi-scale analysis of MSI data, spanning global to fine-grained spatial resolution. Whole-tissue regions can be averaged to support global-level analysis, followed by user-defined spatial segmentation for regional interrogation. High-spatial-fidelity analysis is achieved through HexBin discretization, with the level of spatial fidelity flexibly controlled by the “xbins” parameter, which determines the density of the HexBin grid. (C) SCOPE supports both feature-centric and spatial-centric analyses. Feature-centric analysis enables direct statistical comparison of *m*/*z* features across samples using low-spatial-fidelity data, whereas spatial-centric analysis is performed exclusively after HexBin discretization and conducted at the population level. This includes analyses such as spatial clustering, spatial correlation of signal-change magnitude, and spatial change mapping.

Beyond standard preprocessing, SCOPE operates across 3 spatial scales, whole section, user-defined region analysis, and hexagonal spatial units (HexBins) to balance interpretability with spatial precision in spatially resolved MSI datasets (Fig. 1). SCOPE is implemented as a general spatial alignment and comparison framework that integrates rigid geometric transformations, deformable alignment, and spatial discretization to enable direct, location-matched comparisons across biological replicates using a defined spatial unit (Fig. 1A). In practice, the workflow maps biological sections onto a standardized template shape, achieving high spatial fidelity through sequential co-registration followed by HexBin-based spatial discretization. The template shape may be derived from the average geometry of the cohort or, where appropriate, through analytical geometry standardization based on known or inferred tissue shapes [43]. This design allows SCOPE to be applied to tissues with diverse global geometries and varying degrees of anatomical definition. In the present study, we applied SCOPE to the bovine lens, a tissue characterized by smooth global geometry and the absence of sharply defined internal anatomical boundaries (see Fig. S1). Accordingly, a geometry-assisted approach was used to align individual sections to a common reference shape (Fig. S4a (i) and (ii)) [43]. Each tissue section was first centered and rescaled to ensure spatial comparability [44]. Tilt correction was performed through ICP-guided rigid transformations, aligning corresponding spots between each tissue section and the reference template by optimizing rotation and translation to minimize positional discrepancies while preserving intrinsic tissue morphology (Fig. S4a (iii)). Following those initial rigid transformation steps, sections were deformably aligned to the shared anatomical template using the barycentric deformation model [46]. Corresponding spot pairs guided the deformation, with barycentric weights assigned to each point to determine its contribution to local geometry (Fig. S4a (iv); see the Methods section “High-spatial-fidelity analysis at the HexBin level via SCOPE”). This weighting preserved local spatial topology while compensating for global differences in shape and size, resulting in uniformly shaped, anatomically standardized sections across replicates. Finally, to enable direct quantitative comparison of spatially aligned sections, a universal HexBin mesh was applied to each standardized section [15]. This spatial discretization partitioned the tissue into reproducible spatial units, establishing mesh-to-mesh correspondence across samples. Mapping molecular intensities onto homologous HexBins reduces stochastic pixel-level noise and mitigates artifacts from alignment or sampling differences, enabling robust, spatially matched comparisons at the microdomain level.

First, we benchmarked the spatial co-registration functions of the SCOPE workflow against both our previous rigid-only alignment approach and a no-transformation baseline (lens centering only) [15]. For all alignment strategies, HexBin discretization was applied to enable spatial correlation analysis across the 4 bovine lens exposure groups (see the Methods section “Lens incubation”), each comprising 3 biological replicates, with Pearson correlations calculated between all replicate pairs within each exposure group. Because spatial correlation cannot be meaningfully assessed at the individual-pixel level following alignment, correlations were evaluated consistently at the HexBin level using spatially distinct *m*/*z* features that reflect lens biochemical heterogeneity (Fig. S4b). These features were selected based on their distinct and reproducible spatial distributions across the lens rather than their registration performance. Their complementary molecular distributions across the cortical and nuclear regions provide suitable spatial features for assessing anatomical correspondence between biological replicates. Within this tandem co-registration HexBin framework, barycentric deformable alignment substantially improved spatial reproducibility across bovine lens samples. HexBin-level Pearson correlations for those *m*/*z* features were consistently higher following deformable alignment than after rigid-only alignment or centering without transformation (Fig. S4b). Deformable alignment also reduced interreplicate variability, indicating more accurate anatomical correspondence of molecular signals across lenses. These results demonstrate that the deformable co-registration and HexBin-based spatial units used in the SCOPE pipeline enhanced spatial fidelity when overlaying replicate lens MALDI-MSI datasets. With the validation of our spatial overlay approach, the analysis and visualization tools available in SCOPE can now be demonstrated. In addition to providing high spatial fidelity, SCOPE supports low-fidelity approaches to complement high-resolution insights (Fig. 1B) and a range of analysis and visualization across all spatial scales (Fig. 1C). To contextualize the utility of the HexBin approach, we first present in the bovine lens aging model, whole-section analysis that captures broad tissue-level molecular trends and tissue segmentation into anatomically defined ROIs, which enables biologically interpretable comparisons across regions. These 2 lower-resolution data analysis approaches do not require the spatial alignment and application of HexBin discretization; hence, the nonspatially aligned data are presented.

### Spatial metabolomics mapping reveals region-specific oxidative stress responses in bovine lenses

Since cumulative oxidative stress is a central driver of lens aging and ARN cataract [33], HBO exposure was used to model age-relevant redox perturbations in the lens (Fig. 2A) [33,34]. Bovine lenses were exposed to either HBO or HBN as control. We highlight the changes that a set of molecules primarily involved in lens antioxidant pathways, such as GSH, undergo following oxidative stress (Table S1) [41,42,47]. Ion images of some of these molecules revealed their spatial location and abundance in lenses under both control (HBNc) and oxidative stress (HBOc) conditions (Fig. 2B, Figs. S5 and S6, and Table S2).

**Fig. 2.**
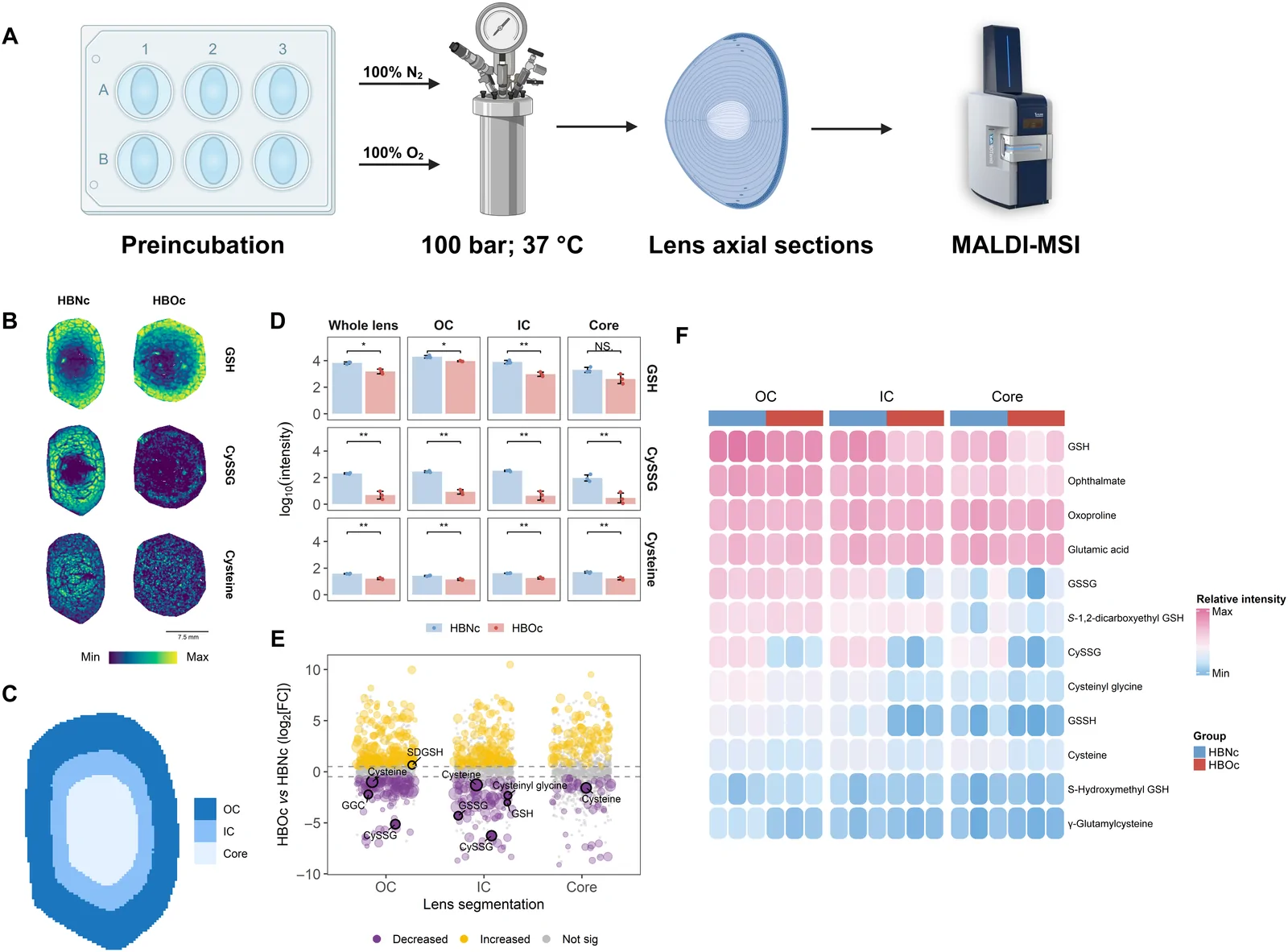
Arbitrary segmentation of bovine lenses revealed region-specific molecular responses to hyperbaric oxygen (HBO)-induced oxidative stress. (A) Whole fresh bovine lenses were incubated in M199 media for 24 h prior to hyperbaric nitrogen (HBN) exposure as control or HBO exposure for oxidative stress. Lenses were maintained at 37 °C during the 15-h exposure at 100 bar of pressure. Lenses were snap frozen to −80 °C, sectioned, and sprayed with matrix according to the methods, before matrix-assisted laser desorption/ionization mass spectrometry imaging (MALDI-MSI) analysis. (B) Ion images of axial sections of bovine lens tissue: top, glutathione (GSH); middle, cysteine-glutathione disulfide (CySSG); bottom, cysteine (CSH). (C) Arbitrary segmentation of the lens into the 3 main regions: outer cortex (OC), inner cortex (IC), and core. (D) Quantification of GSH, CySSG, and CSH, across both the whole lens (left panel) and the individual lens regions (right 3 panels). NS, not significant; *P < 0.05; **P < 0.01. (E) Categorical volcano plot showing metabolites that had a significant fold-change increase (yellow) or decrease (purple) following exposure to HBO *vs* HBN. Fold changes were calculated as the mean intensity across replicates for each group (*n* = 3) within each defined region. In the volcano plot, each point represents an *m*/*z* feature, with the *x*-axis showing the arbitrary segments and the *y*-axis representing the log_2_ fold change. The point size corresponds to the −log_10_ *P* value, with nonsignificant molecules (*P* ≥ 0.05) shown at uniform size. Molecules with *P* values <0.05 and absolute log_2_ fold change >0.5 were considered significantly changed in this plot. (F) Heatmap showing the variability of each metabolite between each biological replicate, lens region, and exposure group. Targeted molecules are listed in the Table S1.

GSH is the primary antioxidant in the lens and is critical for maintaining the specialized redox environment required for transparency [42,48]. Cysteine (CSH), the rate-limiting precursor for GSH synthesis, and cysteine-glutathione disulfide (CySSG), a redox-active conjugate that is thought to act as a storage form of both GSH and CSH, were included to capture complementary aspects of lens redox homeostasis [42]. Representative ion images revealed that control lenses showed higher levels of GSH and CySSG in the cortical regions, whereas CSH was more abundant in the core (Fig. 2B, left). Upon exposure to oxidative stress, the spatial distribution of CySSG was substantially altered, while the distribution of GSH remained largely unchanged. In contrast, CSH exhibited a marked depletion in the lens core following oxidative challenge (Fig. 2B, right). Similar spatial patterns were observed for other metabolites, showing broader region-specific metabolic responses across the lens (Fig. S5). These observations highlight region-specific vulnerabilities in the lens antioxidant network following acute treatment with HBO.

To further quantify the spatial variation of metabolites described above, we arbitrarily segmented the lens sections into concentric anatomical zones, namely, the outer cortex (OC), inner cortex (IC), and core, based on radial distance from the lens center (Fig. 2C and Fig. S7) [15]. Analyses were performed at both the whole-lens and segmented-lens levels (*n* = 3 lens sections per treatment group), which allowed us to statistically test for quantitative differences of signal levels between control (HBNc) and oxidative stress (HBOc) samples. Whole-lens quantification revealed a significant exposure-associated decrease in GSH, CySSG, and CSH (Fig. 2D), although the magnitude of these changes varied across different regions (Fig. 2E), and between biological replicates (Fig. 2F). Therefore, arbitrary segmentations provided a degree of spatial interpretability by capturing broad regional metabolic patterns; however, higher-resolution spatial information intrinsic to the MSI data was averaged within each region and consequently lost (Fig. S8), obscuring fine-scale metabolic gradients and microdomain-level heterogeneity within the lens. To overcome these limitations and potentially reveal spatially confined lens antioxidant changes in response to oxidative stress, we applied the SCOPE pipeline to HBOc and HBNc samples in the first instance. Lens section data were co-registered to a standardized lens geometry and discretized into spatial HexBin (as described previously), with GSH shown as a representative example of the resulting spatial maps (Fig. 3A). Average molecular intensities per HexBin were then subjected to dimensionality reduction using UMAP, which distilled the high-dimensional MSI data into a 2-dimensional representation that preserves local neighborhood relationships while facilitating visualization of broader relationships among spectra [49]. Projection of the HexBin *m*/*z* profiles into this reduced space (Fig. 3B) revealed clear treatment-driven separation, indicating that oxidative stress produced distinct molecular signatures. While UMAP highlighted overall treatment-driven separation, additional clustering was required to delineate finer metabolic subgroups. Accordingly, *k*-means clustering was performed in the reduced molecular space [50]. This data-driven approach allowed us to partition the data based on intrinsic molecular similarity rather than predefined anatomical zones. The resulting cluster assignments were then mapped back to tissue coordinates, producing intuitive spatial maps that revealed regionally distinct metabolic domains across treatment conditions (Fig. 3C to E). In contrast to our arbitrary segmentation (Fig. S9), the data-driven approach resolved molecularly distinct clusters arranged in broadly concentric patterns that were consistent with the known anatomical organization of the bovine lens (OC, IC, and core) [42,51–53] while capturing additional spatial heterogeneity beyond the predefined anatomical regions. Moreover, this approach also captured a clear anterior–posterior asymmetry in these divisions, with regional boundaries more closely spaced in the posterior lens than in the anterior region (Fig. 3D), suggesting spatially distinct metabolic organization along the anterior–posterior axis. This close alignment between molecular clusters and anatomical structure demonstrates that the computational pipeline captures spatially coherent biochemical variation and preserves biologically meaningful gradients, establishing a robust foundation for subsequent quantitative spatial analysis. Projecting the same dimension back onto the original lens coordinates revealed a pronounced, spatially distinct decrease in the fold change of GSH concentrated in the IC (Fig. 3F and G and Fig. S10). With the higher spatial detail provided by the molecular maps at the HexBin level, it became evident that the depletion within the IC was not uniform. Specifically, as visualized by HexBin-based signal-change maps, lens fiber cells in the anterior lens showed a markedly greater reduction than those located in the posterior region, indicating region-specific susceptibility to oxidative stress (Fig. 3H and I). In contrast, the lens core exhibited no significant change in GSH abundance (Fig. 3I), consistent with arbitrary segmentation results (see Fig. 2D). Although the fold change in GSH at the lens periphery was less pronounced than in the IC, the absolute decrease was larger (Fig. 3J), reflecting the higher baseline antioxidant concentration in the periphery and its greater utilization under oxidative challenge. The visualization of these absolute- and relative-change maps is possible as a direct result of our SCOPE-enabled data overlay pipeline.

**Fig. 3.**
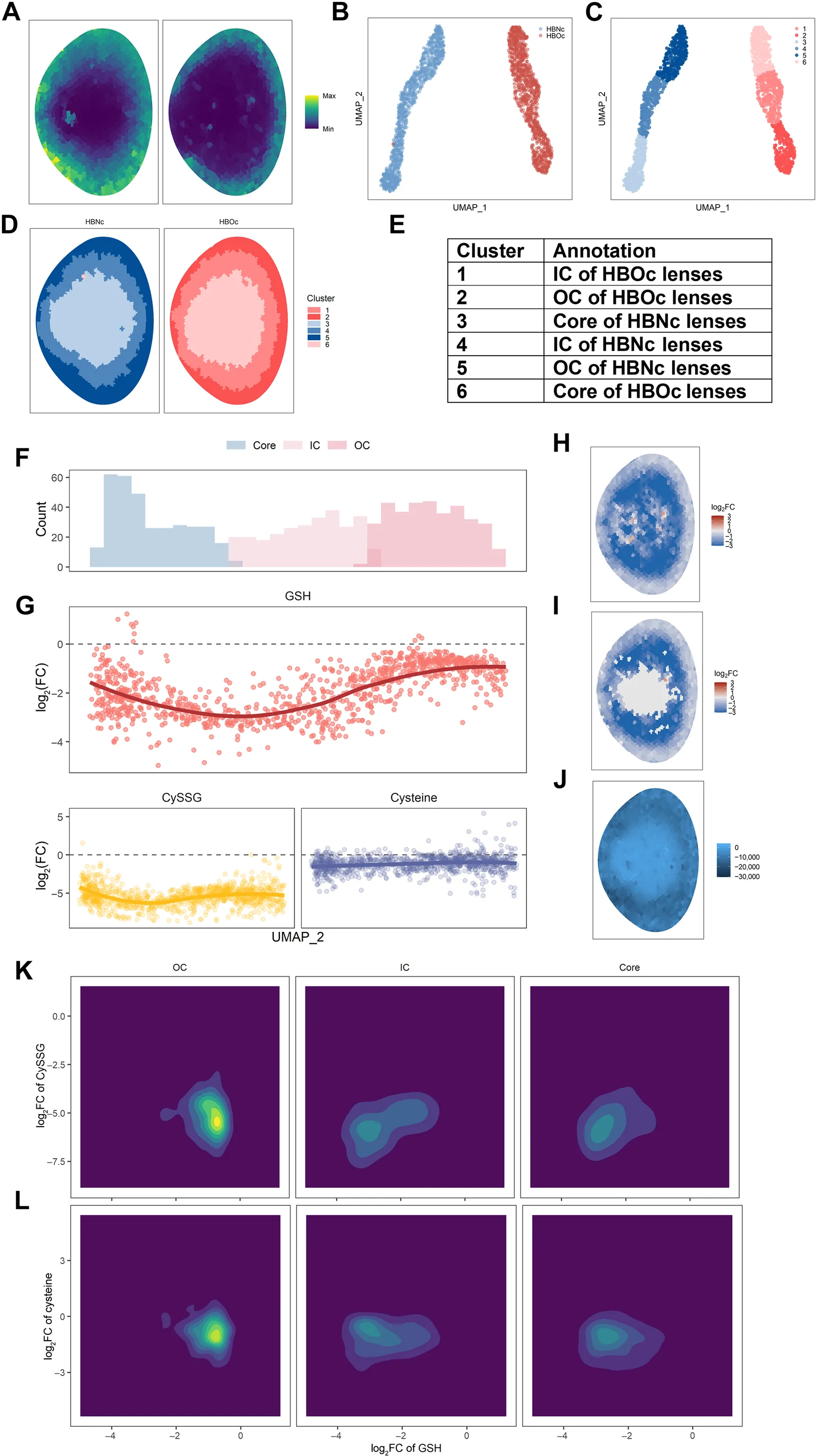
Spatial metabolomics mapping reveals region-specific oxidative stress responses in the bovine lens. (A) Representative ion images of glutathione (GSH) after co-registration (left, HBNc; right, HBOc). HBNc, hyperbaric nitrogen without *N*-acetylcysteine amide (NACA); HBOc, hyperbaric oxygen without NACA. (B) Application of uniform manifold approximation and projection (UMAP) to HexBin-level matrix-assisted laser desorption/ionization mass spectrometry imaging (MALDI-MSI) data derived from averaged ion intensities across biological replicates (*n* = 3 per exposure group). The uniform manifold approximation and projection (UMAP) embedding projects the high-dimensional spatial metabolomics data into a 2-dimensional space, enabling visualization of molecular clustering and separation between oxidative-stressed lenses (HBOc, red) and control lenses (HBNc, blue). (C) The subsequent spatial *k*-means clustering map displays the localized distribution of these clusters based on HexBins. Each color corresponds to a specific cluster, with the legend on the right denoting 6 clusters. (D) The clusters were then projected to standardized lens geometry for annotations. UMAP largely separated the exposure effect (UMAP 1) and lens spatial gradients (UMAP 2). (E) Annotations of the lens segmentations in (D). (F) HexBin counts for each spatial segmentation are shown using HBNc annotations as the reference, along the spatial axis of the UMAP embedding (UMAP 2). (G) Fold change (FC) (HBOc *vs* HBNc) for each HexBin along the UMAP spatial axis for representative molecules. (H) FC map of GSH across HexBins on the standardized lens geometry. The fiber cells in the inner cortex (IC) that are closer to the posterior region exhibit lower FC than those closer to the anterior. (I) FC map of GSH across HexBins on the standardized lens geometry after multiple-testing correction. *P* values were adjusted using the Benjamini–Hochberg false discovery rate (FDR) procedure across all tested *m*/*z* features and HexBins, and only HexBins with an FDR-adjusted *P* value <0.1 are displayed. (J) Absolute differences in average GSH intensities across HexBins on the standardized lens geometry (*n* = 3). (K and L) Spatial covariation of oxidative-stress-induced metabolic perturbations across the lens. HexBin-wise correlations of oxidative-stress-induced FC magnitudes between GSH and cysteine-glutathione disulfide (CySSG) (K) and GSH and cysteine (L) are shown for different lens segmentations. For each region, the FC for each metabolite was calculated independently within each HexBin (*n* = 3), and the magnitude of these changes was compared across all HexBins within that region.

An additional feature of the pipeline allows a HexBin-level fold-change magnitude correlation analysis, which enables the quantification of how 2 molecules covary across the tissue (Fig. 3K and L and Fig. S11). Although GSH, CySSG, and CSH were globally depleted following oxidative stress (see Fig. 2D), their spatial relationships differed markedly across anatomical regions. Across-region HexBin-level fold-change magnitude correlation analysis revealed distinct coupling patterns between GSH and its related thiol-containing molecules. For both CySSG and CSH, the OC exhibited a negative correlation slope, indicating that pixels with stronger GSH depletion did not experience proportional decreases. This decoupling suggests that the OC underwent a more heterogeneous oxidative response, where local GSH loss was not strictly dictated by CySSG availability. In contrast, the IC and core displayed positive correlation slopes, suggesting that the magnitude of GSH loss spatially covaried with changes in CSH and CySSG across HexBins. This coordinated response suggests that these measured thiol-containing metabolites respond in a linked manner within deeper lens regions, consistent with a more homogeneous redox perturbation compared with the OC. Together, these results underscore that redox responses to oxidative stress are spatially stratified across the lens, with the OC exhibiting distinct metabolic decoupling compared with deeper regions. This is potentially due to the ability of fiber cells in the OC to synthesize GSH, and higher levels of NADPH and enzyme activity that supports the regeneration of GSH from its oxidized form, GSSG [54].

With the efficacy of our spatial alignment demonstrated and the ability of HexBin-based analysis to map the spatial response of lens antioxidants to an HBO stress established, we then used this approach to spatially resolve the effects of pharmacological mitigation of the HBO stress. Using our pipeline, we compared sections from lenses (*n* = 3) pre-treated with NACA, a lipophilic CSH analog with enhanced membrane permeability, with the HBNc and HBOc lens MSI data at multiple spatial scales, i.e., first in whole section and regional analysis (using nonspatially aligned sections) and then at the HexBin level (using spatially aligned data).

### Spatially resolved effects of NACA on lens redox metabolism under oxidative stress

Therapeutic elevation of lenticular GSH to protect against oxidative stress is inherently challenging due to anatomical and intracellular barriers that restrict GSH delivery to deeper lens regions [48], prompting interest in lipid-permeable CSH-based analogs. NACA is thought to support GSH synthesis by increasing the availability of intracellular CSH [55–57], which is subsequently used in conjunction with glycine and glutamate in the synthesis of GSH, via the action of GSH synthetase [58]. Thus, control or oxidatively stressed lenses preincubated with NACA (HBNn and HBOn, respectively; Table S2) were assessed for the presence and distribution of key GSH-related metabolites in comparison to lenses not preincubated in NACA (HBNc and HBOc).

Untargeted analysis of MSI data from (nonspatially aligned) whole lens sections revealed that substantial metabolite changes were observed following NACA incubation, with a subset of features commonly perturbed across conditions and others exhibiting condition-specific responses (Fig. 4A). To enable direct comparison with previous microdissection studies [11,55], spatial patterns were first summarized using arbitrarily defined regions corresponding to the OC, IC, and core (Fig. 4B and Fig. S12). Under HBN, NACA incubation (HBNn) resulted in increased signal intensity for CSH in all 3 lens regions compared to the control (Fig. 4B, right), but this signal was reduced following HBO exposure (HBOn). In contrast, GSH levels were not increased by NACA under basal conditions (HBNn, Fig. 4B, left). The CySSG signal was increased in all 3 lens regions following NACA incubation (HBNn) compared to that in the control (HBNc, Fig. 4B, middle) but was reduced under HBO exposure regardless of NACA treatment. However, the reduction in CySSG levels within the OC was less pronounced in NACA-incubated lenses (HBOn) than in HBO control lenses (HBOc).

**Fig. 4.**
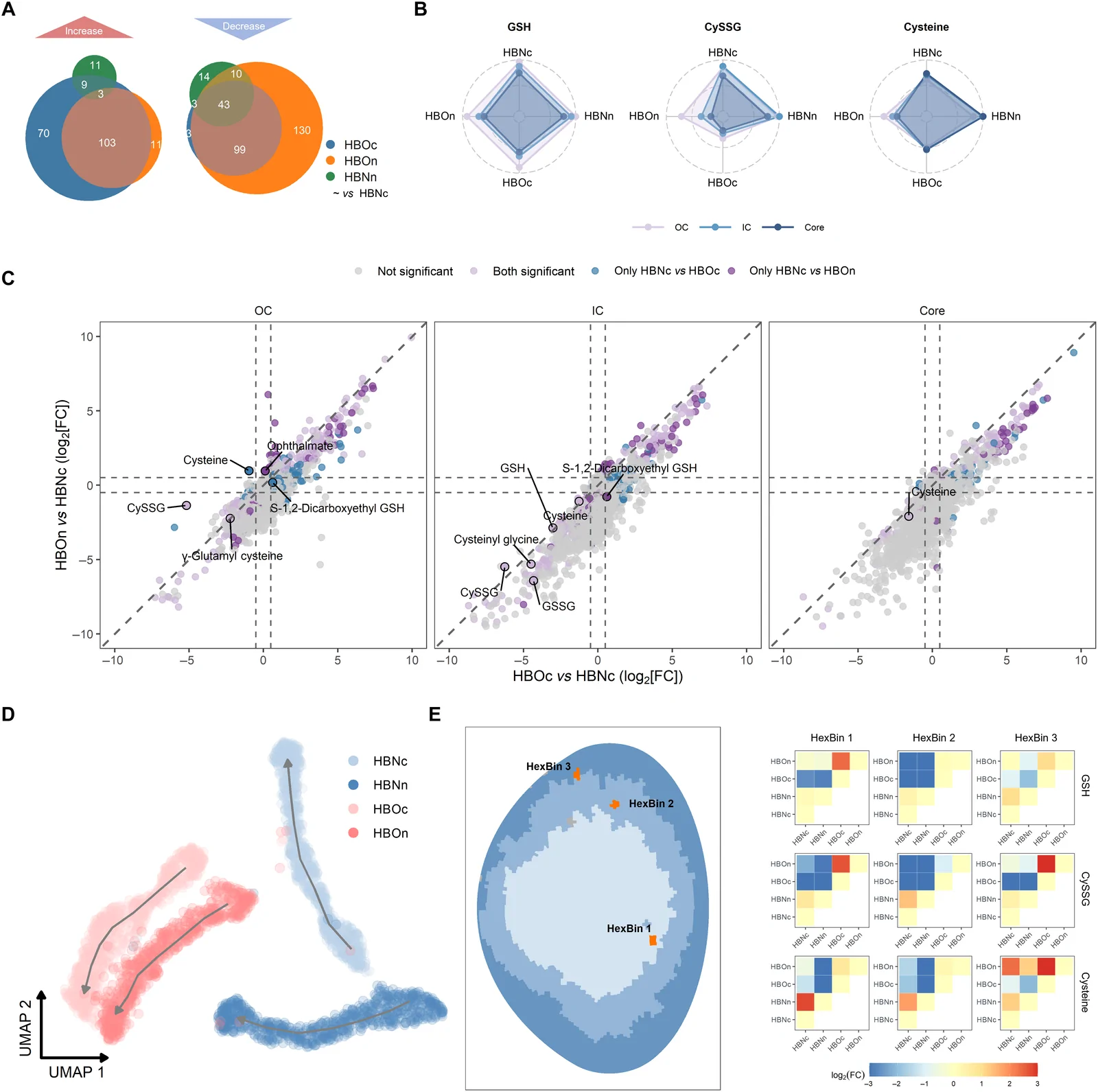
SCOPE facilitates the analysis of drug effects from global to fine spatial detail. (A) Average spectra of whole lens areas were calculated and statistically analyzed for all exposure groups (*n* = 3). Venn diagrams revealing the number of significantly increased (left) and decreased molecules in every group relative to the HBNc group (*P* value < 0.05). HBNc, hyperbaric nitrogen without *N*-acetylcysteine amide (NACA). (B) Radar plots depict the mean log_10_-transformed regional intensity of metabolites within predefined lens regions—outer cortex (OC), inner cortex (IC), and core—across all exposure groups (*n* = 3). (C) Fold change (FC)/FC plot comparing metabolic responses under HBO + NACA (*Y*-axis; FC relative to HBN) against HBO alone (*X*-axis; FC relative to HBN), stratified by lens region (*n* = 3). Molecules with *P* values <0.05 and absolute log_2_ FC >0.5 were considered significantly changed in this plot. HBO, hyperbaric oxygen; HBN, hyperbaric nitrogen. (D) The uniform manifold approximation and projection (UMAP) embedding demonstrates metabolic gradients extending from the lens periphery to the core, revealing a distinctive metabolic architecture and a clear exposure-dependent effect. (E) Heatmap of FCs in representative HexBins for glutathione (GSH), cysteine-glutathione disulfide (CySSG), and cysteine (CSH), showing that NACA mitigates HBO-induced oxidative stress in a spatially dependent manner.

To directly visualize the impact of NACA incubation on metabolites altered by oxidative stress, fold changes in HBO-exposed lenses were plotted against fold changes in HBO + NACA-incubated lenses across all arbitrarily defined regions (Fig. 4C). The HBO-induced depletion of GSH was not mitigated by NACA in any region, indicating that any NACA-induced GSH synthesis was not sufficient to mitigate GSH utilization in response to oxidative stress. CSH was significantly depleted in the OC following HBO exposure; however, this reduction was not significant when lenses were supplemented with NACA (Fig. 4C, left). The decrease in γ-glutamyl cysteine in the OC despite NACA incubation suggests that supplementation with CSH alone is not sufficient to continually drive GSH synthesis [59], as other precursors, such as γ-glutamyl cysteine, may then become rate-limiting steps in synthesis. Ophthalmate, a tripeptide related to GSH, was significantly increased in the OC following NACA incubation. As a non-redox-active GSH analog, ophthalmate accumulation serves as a metabolic marker of an activated antioxidant response under oxidative stress, rather than a direct contributor to redox defense [60]. Although CySSG levels fell significantly in the OC of both HBOc and HBOn lenses, the magnitude of this decline (fold change) was modest following NACA incubation. Furthermore, *S*-1,2-dicarboxyethyl GSH (SDGSH) was increased in the OC following HBO exposure (HBOc; Fig. 4C, left), consistent with previous findings [42], but decreased in the IC (Fig. 4C, middle). The increase in SDGSH in the OC likely reflects an increase in l-malate and/or fumarate output from the citric acid cycle, which is active in this lens region [42]. Interestingly, GSSG was more significantly reduced in the IC of HBOn lenses than in the IC of HBOc lenses (Fig. 4C, middle).

Finally, we utilized the additional spatial information of our spatially aligned HexBin approach to visualize and compare all exposure groups by 2-dimensional UMAP embedding (Fig. 4D). Annotations of the HexBins to specific exposure groups indicated that NACA exerts some metabolic effect across each of the different lens regions. In addition, the arrow within each UMAP cluster indicates the metabolic gradients from the lens periphery to the lens cores. Within each exposure condition, NACA-incubated (HBNn and HBOn) and control (HBNc and HBOc) samples form closely related subclusters, demonstrating that NACA incubation influences the metabolome in all lens regions but does not fully protect the lens from the primary effects of HBO-induced oxidative stress. These UMAP patterns show that oxidative stress is the dominant driver of metabolic divergence, with NACA exerting a partial modulatory effect.

To further investigate the local difference at a HexBin level, a fold-change heatmap was used to visualize NACA effects on specific molecules across any 2 exposure groups for any HexBin. This analysis showed that NACA could generally reduce the HBO-induced depletion of antioxidants across the whole lens (Fig. 4E). For example, in the OC and core (HexBins 3 and 1, respectively), GSH, CySSG, and CSH were all increased in HBOn lenses compared to those in HBOc lenses. While the quantity of these metabolites was lower in HBOn lenses than baseline (HBNc), these results highlight the potential function of NACA in protecting the lens against the effects of oxidative stress.

In summary, by using co-registration of datasets to a common spatial reference, UMAP and *k*-means algorithms to resolve continuous variation, and HexBin-based quantification encompassing multiple biological replicates, we were able to visualize subtle metabolic gradients, capture asymmetries of anterior and posterior lens biochemistry, identify previously unrecognized microdomains of oxidative stress response, and localize the response of the aging lens to a therapeutic intervention. This highlights the strength of high-spatial-fidelity analysis in revealing fine-scale molecular patterns that may have important physiological implications for lens transparency and aging.

## Discussion

In this study, we developed a workflow to co-register and analyze spatial metabolomics MALDI-MSI datasets across biological replicates and treatments. This workflow addresses 2 long-standing obstacles in comparative MSI analysis: intersample variability in tissue size and morphology and the absence of consistent pixel-to-anatomy correspondence inherently arising from raster-based acquisition. Variability in tissue shape and orientation, combined with raster-based acquisition, prevents pixels from corresponding to the same anatomical locations across samples, rendering direct pixel-wise comparison infeasible. To overcome this limitation, SCOPE deploys a deformable co-registration algorithm in tandem with a universal spatial HexBin discretization, replacing native pixel coordinates with a shared anatomical spatial framework. Within this framework, each HexBin represents a spatial unit that is anatomically consistent across samples, enabling aligned spatial quantification across multiple replicates at high spatial fidelity.

This approach differs from existing MSI frameworks that rely on whole-tissue averaging, coarse anatomical segmentation, or unaligned pixel-level statistics, which either discard spatial context or lack a mechanism to establish anatomical correspondence across samples [6,9,18,20–23,26,39,40,61,62]. Analyses conducted at coarser spatial scales can yield interpretable summaries of general molecular composition, whereas progressively finer scale increases spatial specificity and sensitivity to local molecular heterogeneity. SCOPE therefore benefits from explicitly structuring analyses across multiple spatial scales to balance biological interpretability with spatial fidelity. While we acknowledge that HexBin discretization introduces controlled spatial aggregation, and therefore a loss of effective spatial resolution, by combining neighboring pixels, this aggregation is a deliberate and necessary process that enables anatomically meaningful comparisons across multiple tissue sections. Importantly, spatial fidelity within SCOPE is linked to the underlying imaging resolution. Finer raster sampling translates into finer spatial granularity within the HexBin framework when the number of pixels per HexBin remains largely unchanged. Consistent with this design, spatial Pearson correlations between aligned biological replicates demonstrate sufficient agreement for meaningful HexBin-level comparisons at the population level (Fig. S4b). In this setting, correlation reflects preservation of relative spatial patterns rather than exact pixel-wise intensity concordance, which is neither expected nor required given biological variability and the somewhat stochastic nature of spectral acquisition in MALDI-MSI. Residual spatial variance is therefore anticipated and is more likely to reflect biological heterogeneity and technical noise than systematic misalignment; any remaining unsystematic misregistration would increase variance within HexBins rather than bias inference. By drawing on consistent spatial trends across bins at the population level, SCOPE supports inference without treating any individual HexBin as ground truth. These design choices position SCOPE as a bridge between high-resolution MSI data acquisition and biologically meaningful multisample spatial analysis, capturing fine-scale molecular heterogeneity without resorting to pixel-level comparisons.

We demonstrated the distinct advantage of high-spatial-fidelity analysis in investigating the local metabolic responses of the lens to oxidative stress and NACA supplementation. In the first instance, control and treated lenses exhibited asymmetry along the visual axis (i.e., anterior–posterior), which was not reflected in an arbitrary segmentation approach. While this patterning was observed in the relatively young bovine lenses used in the present study, a similar asymmetry has been detected previously when mapping GSH uptake in a young (26-year-old) human lens [63]. That is, the region defined as the lens core was closer to the posterior surface than the anterior surface. In addition, we identified anterior–posterior asymmetry in the response of the lens to oxidative stress, with fiber cells proximal to the posterior pole exhibiting reduced responsiveness. This is likely due to the pronounced asymmetry of the lens core and that antioxidant molecules such as GSH are already at low levels in the core relative to the cortex. Asymmetric metabolic patterns have previously been reported in the lens using MALDI-MSI ion images. However, these were due to glucose metabolism pathways and related enzymatic activity, rather than the antioxidant system and oxidative stress response. In addition, those observations were largely qualitative and lacked anatomically aligned, high-resolution quantification across biological replicates [38]. Our use of the SCOPE pipeline here showed that individual lens regions respond differently to oxidative stress, consistent with previous reports linking regional oxidative stress to tissue-wide damage and the development of ARN cataract [64–66].

Applying this framework to lenses supplemented with NACA, we detected that NACA increased CSH levels in the superficial fiber cells, whereas no significant increase was observed in deeper fiber cells. While this aligns with previous studies that used tissue microdissection into lens cortical and core regions [55], SCOPE increases the specificity of this spatial analysis, resolving additional information at the individual HexBin level. Interestingly, CySSG was also increased following NACA treatment and partially depleted following HBO treatment, suggesting that it could be utilized in each lens region and potentially add to the pools of CSH and GSH in response to oxidative stress. These region-specific responses highlight SCOPE’s ability to capture subtle, localized metabolic changes and underscore the importance of preserving spatial context in structured tissues, where averaging across regions can obscure functionally relevant microdomains. By maintaining anatomical correspondence, SCOPE enables robust, high-fidelity detection of these subtle variations, establishing a generalizable framework for comparative molecular imaging.

We acknowledge that these findings are based on a relatively small number of biological replicates (*n* = 3 per group), which can limit the statistical power of the present study. The observed spatial pattern was consistently reproduced by both arbitrary ROI-based segmentation and the higher-spatial-fidelity HexBin analysis, yielding concordant results. Furthermore, the spatial co-registration framework was independently evaluated using spatial Pearson correlation analysis, which demonstrated improved anatomical correspondence following deformable registration compared with rigid-only and no-transformation approaches. Although additional biological replicates would further strengthen these findings, the agreement between complementary analytical approaches and the improved spatial reproducibility provide support for the observed regional differences.

Although demonstrated here in the context of ocular lens biology, the barycentric deformation model was selected because it provides a smooth geometric interpolation of interior pixel locations from corresponding boundary landmarks. As the transformation is defined entirely by the spatial relationships between matched boundary points, it is well suited to the crystalline lens, whose smooth global geometry and lack of large internal discontinuities enable reliable boundary-guided registration. In addition, the SCOPE framework is not restricted to the barycentric deformation model. Its tandem co-registration and HexBin strategy is compatible with a range of deformable registration approaches, including landmark- and control point-based free-form deformation methods that register tissue sections to curated anatomical atlases [67]. Such template-based co-registration strategies are particularly well suited to tissues with well-characterized internal anatomical structures and established reference atlases. For these tissues, comparison with independent histological references or anatomical atlases could also provide further validation of the accuracy of internal registration. When combined with our pipeline, they would form a comprehensive framework that enhances spatial reproducibility, improves quantitative reliability, and deepens the interpretability of MALDI-MSI data in anatomically complex tissues such as the brain and lung [67,68]. In contrast, tissues characterized by extensive structural heterogeneity or invasive remodeling, such as solid tumors, remain challenging for any template-based registration strategy because establishing anatomically homologous spatial correspondence across samples is inherently difficult. In these settings, histology-guided or computationally defined region-based analyses may remain the more appropriate approaches.

In conclusion, SCOPE addresses a critical need in the MSI field, providing an open-source workflow for anatomically aligned, high-spatial-fidelity analysis of MALDI-MSI datasets across biological replicates. Using SCOPE, we revealed microdomain-level metabolic heterogeneity and anterior–posterior asymmetries in *ex vivo* bovine lenses and quantified region-specific responses to oxidative stress and therapeutic intervention. The potential of this framework allows the integration of multiple MALDI-MSI datasets and extension to other imaging modalities, including MRI and elemental imaging. This flexibility positions SCOPE as a scalable platform for spatially resolved multi-omics across diverse tissues, enabling mechanistic insights in a range of biological and pathological contexts.

## Data Availability

The raw .imzML files have been deposited in Metabolomics Workbench (Study ID: ST004748; doi: 10.21228/M83275) [69]. The SCOPE source code and example dataset are available from https://github.com/Dingchang-UoA/SCOPE (release 1.0.0). Installation and analysis instructions are provided in the repository.
