# Supplementary material for: SCOPE: A Spatial Co-registration and Omics Pipeline for Comparative Mass Spectrometry Imaging Data Analysis
Source: Comput Struct Biotechnol J. 2026 Oct 26;2026issue-1(1):0237. doi: 10.34133/csbj.0237 (PMC13696801; doi:10.34133/csbj.0237)
Supplement: Supplementary file 1 [file csbj.0237.f1.docx]

# **SUPPLEMENTARY MATERIALS**

Supplementary Figure 1 A lens schematic.

The lens is enveloped by a thin, transparent capsule (green). A single layer of cuboidal epithelial cells (yellow) covers its anterior surface. The lens fibre cells, which make up the bulk of the lens cell mass, can be broadly divided into the outer cortex (light pink), inner cortex (pink), and core (purple), depending on their relative depth toward the centre of the lens.

Supplementary Figure 2 UMAP-based identification of on-/off-tissue regions.

**a** An optical image of the bovine lens axial section used for MALDI-MSI. **b** K-means clustering of features after UMAP dimensionality reduction identified five distinct spatial clusters. **c** Spatial projection of the UMAP clusters onto the lens section reveals that clusters 1 and 5 correspond to boundary and off-tissue regions. Clusters 3, 4 and 2 approximately represent the lens outer cortex (OC), inner cortex (IC), and core. **d** A binary mask showing the excluded off-tissue pixels (black) prior to downstream analysis.

Supplementary Figure 3 Identification of on-/off-tissue regions of all runs.

**a** A binary mask showing the excluded off-tissue pixels (black) prior to downstream analysis. **b** Mean spectra of the off-/on-tissue regions. Off-tissue regions have lower peak intensities comparing with the on-tissue regions.

Supplementary Figure 4 Barycentric deformation model to co-register the lens to a standardised lens geometry.

**a** *i* Standardised geometry of bovine lenses generated using a method described previously (43), providing a reference geometry for spatial alignment*.* *ii* A representative geometry of the lens sections for MALDI-MSI*.* *iii* Point correspondence established via iterative closest point (ICP)-based spot matching. The red points indicate locations within the lens model and should be shifted outward, whereas the blue points denote locations outside the lens and should be shifted towards the model. *iv* Pixels within the experimental sections are relocated according to their barycentric weights, which are defined by the trigonometric relationships between two adjacent points on the edge and the centre (0,0). **b** Boxplots show Pearson correlation coefficients for anatomically distinct *m/z* features (288.1209, 306.0773, 422.0885, and 611.1461) calculated at the HexBin level across biological replicates under three alignment strategies: no transformation (centring only), rigid transformation, and barycentric deformable model. For all these anatomically distinct features, the deformable alignment consistently yields higher correlations and reduced dispersion compared with rigid-only and no-transformation approaches. Deformable registration enabled anatomically homologous spatial correspondence across replicates, forming the basis for reproducible population-level spatial analysis. Points represent Pearson correlations between pairs of biological replicates within each exposure group (three pairs per group across four groups; 12 correlations per metabolite and alignment method). These features were selected because they exhibit distributions that demarcate the different anatomical regions of the lens. For example, m/z 288.1209 (tentatively annotated as ophthalmate, OPH) is predominantly localised to the cortical regions, whereas m/z 306.0773 (GSH) is enriched in the outer cortex. Similarly, m/z 422.0885 (S-1,2-dicarboxyethyl GSH, SDGSH) and m/z 611.1461 (GSH disulfide, GSSG) both display reproducible concentric spatial patterns with clear contrast between the lens core and peripheral regions.

Supplementary Figure 5 Ion images of axial sections from all biological replicates of bovine lenses. The spatial distributions of glutathione (GSH) (top row), cysteine–glutathione disulfide (CySSG) (middle row), and cysteine (CSH) (bottom row) are shown for lenses incubated under hyperbaric nitrogen (HBN; left, n = 3) and hyperbaric oxygen (HBO; right, n = 3). Ion images are displayed using the same colour scale for each metabolite to facilitate comparison across biological replicates. Scale bar = 7.5 mm.

Supplementary Figure 6 Spatial distributions of glutathione (GSH)-related molecules.

Representative ion images illustrate the distribution of GSH-related metabolites (scale bar, 7.5 mm) in control (HBNc) and oxidatively stressed lenses (HBOc).

Supplementary Figure 7 Statistical comparisons of molecular abundances at the whole-lens level and across spatial arbitrary segmentations. Bars show mean ± SD; points represent individual biological replicates (n = 3 per group).

OC: outer cortex; IC: inner cortex; core) using t-tests (n = 3, NS = not significant, *P < 0.05, **P < 0.01, ***P < 0.001).

Supplementary Figure 8 The distribution of the pixels within each lens segmentation.

Violin plots depict the variation in molecular intensities of the assessed metabolites across spatially defined regions: OC, IC, and core. The distributions reveal that molecular intensities are unevenly distributed within each region, as evidenced by the presence of bimodal patterns in the violin plots. This indicates that averaging high-resolution MSI data within these arbitrarily defined regions obscures finer spatial heterogeneity.

Supplementary Figure 9 PLS-DA assesses spatial segmentation performance.

**a** The region clusters generated using arbitrary segmentation show substantial overlap, indicating limited separation. **b** Clusters derived from data-driven segmentation are more distinct, suggesting that arbitrary segmentation can obscure underlying spatial molecular patterns due to the large degree of cluster overlap. **c** VIP plot showing the top 5% of *m/z* features identified by PLS-DA based on data-driven segmentation, including GSH, GSSG, and CySSG, which are known from previous studies to exhibit spatial differences (42,47).

Supplementary Figure 10 Data-driven clustering show spatial molecular change along UMAP spatial dimension.

Supplementary Figure 11 HexBin-wise correlation of fold-change magnitudes between molecular pairs across lens regions.

For each molecular pair, fold-change magnitudes were compared within corresponding HexBins. Lower panels show the joint distribution of HexBin-wise fold-change magnitudes, visualized as 2D density plots. Upper panels report the corresponding Pearson correlation coefficients calculated across all shared HexBins. Analyses were performed separately for each region, enabling region-specific comparison of molecular co-variation patterns.

Supplementary Figure 12 Radar plots depict the mean fold change of metabolites within predefined lens regions—outer cortex (OC), inner cortex (IC), and core across all exposure groups (n=3).

| Metabolite | Abbreviation | Formula | Adduct | Theoretical mass  (*m/z*) | Observed Mass  (*m/z*) | Absolute mass error (ppm) | Validation | Isotopic Scoring |
| --- | --- | --- | --- | --- | --- | --- | --- | --- |
| Glutathione | GSH | C_10_H_17_N_3_O_6_S | [M-H]^-^ | 306.0765 | 306.0773 | 2.61 | On-tissue MS/MS | 4.60 |
| Cysteine-glutathione disulfide | CySSG | C_13_H_22_N_4_O_8_S_2_ | [M-H]^-^ | 425.0806 | 425.0815 | 2.12 | LC-MS/MS | 3.46 |
| Cysteinyl glycine |  | C_5_H_10_N_2_O_3_S | [M-H]^-^ | 177.0339 | 177.0349 | 5.65 |  | 1.92 |
| Glutathione disulfide | GSSG | C_20_H_32_N_6_O_12_S_2_ | [M-H]^-^ | 611.1447 | 611.1461 | 2.29 | LC-MS/MS | 2.67 |
| S-1,2-dicarboxyethyl GSH | SDGSH | C_14_H_21_N_3_O_10_S | [M-H]^-^ | 422.0875 | 422.0885 | 2.37 | LC-MS/MS | 3.87 |
| Glutamic acid |  | C_5_H_9_NO_4_ | [M-H]^-^ | 146.0459 | 146.0466 | 4.79 |  | 4.57 |
| Oxoproline |  | C_5_H_7_NO_3_ | [M-H]^-^ | 128.0353 | 128.0360 | 5.47 |  | 3.99 |
| γ-glutamyl cysteine | GGC | C_8_H_14_N_2_O_5_S | [M-H]^-^ | 249.0551 | 249.0582 | 12.45 |  | 2.12 |
| S-sulfanyl-GSH | GSSH | C_10_H_17_N_3_O_6_S_2_ | [M-H]^-^ | 338.0486 | 338.0491 | 1.48 | LC-MS/MS | 1.30 |
| S-hydroxymethyl GSH | SHGSH | C_11_H_19_N_3_O_7_S | [M-H]^-^ | 336.0871 | 336.0854 | 5.06 | LC-MS/MS | 3.62 |
| Cysteine | CSH | C_3_H_7_NO_2_S | [M-H]^-^ | 120.0125 | 120.0127 | 1.67 |  | 2.09 |
| Ophthalmate | OPH | C_11_H_19_N_3_O_6_ | [M-H]^-^ | 288.1201 | 288.1209 | 2.78 |  | 3.99 |

Supplementary Table 1 Identification of glutathione (GSH)-related metabolites of interest.

Theoretical masses were calculated from empirical formulae, and delta values represent the differences between theoretical and observed masses. Assignment of m/z values was based on accurate mass matching (mass error <15 ppm), with additional confidence assessed through isotope pattern matching using HiT-MAP scores (6) and supported, where available, by previous LC-MS/MS (42) or on-tissue (41) MS/MS validation.

| Abbreviation | Exposure |
| --- | --- |
| HBNc | Pre-incubate for 24 h in Gibco Medium 199, then expose to hyperbaric nitrogen at 100 bar for 15 h at 37 °C. |
| HBOc | Pre-incubate for 24 h in Gibco Medium 199, then expose to hyperbaric oxygen at 100 bar for 15 h at 37 °C. |
| HBNn | Pre-incubate for 24 h in Gibco Medium 199 supplemented with 10 mM NACA, then expose to hyperbaric nitrogen at 100 bar for 15 h at 37 °C. |
| HBOn | Pre-incubate for 24 h in Gibco Medium 199 supplemented with 10 mM NACA, then expose to hyperbaric oxygen at 100 bar for 15 h at 37 °C. |

**respective media were replenished prior to hyperbaric exposure*

Supplementary Table 2 Abbreviations of the exposure groups in this study.
